# Supplementary material for: Melanoma Clinical Decision Support System: An Artificial Intelligence-Based Tool to Diagnose and Predict Disease Outcome in Early-Stage Melanoma Patients
Source: Cancers (Basel). 2023 Apr 6;15(7):2174. doi: 10.3390/cancers15072174 (PMC10093614; doi:10.3390/cancers15072174)
Supplement: Supplementary file 1 [file cancers-15-02174-s001.zip › cancers-2234461-supplementary.pdf]

cancers

MDPI

Article

Melanoma Clinical Decision Support System: An Artificial Intelligence-Based Tool to Diagnose and Predict Disease Outcome in Early-Stage Melanoma Patients

Jose Luis Diaz-Ramón [1,2,†], Jesus Gardeazabal [1,2,†], Rosa Maria Izu [2,3,†], Estibaliz Garrote [4,5], Javier Rasero [6], Aintzane Apraiz [2,5], Cristina Penas [2,5], Sandra Seijo [7], Cristina Lopez-Saratxaga [4], Pedro Maria De la Peña [7], Ana Sanchez-Diaz [2,3,], Goikoane Cancho-Galan [2,8], Veronica Velasco [1,9], Arrate Sevilla [2,10], David Fernandez [11], Iciar Cuenca [7], Jesus María Cortes [2,5,12], Santos Alonso [10], Aintzane Asumendi [2,5,*] and María Dolores Boyano [2,5,*]

1 Dermatology Service, Cruces University Hospital, 48903 Barakaldo, Spain; joseluis.diazramon@osakidetza.eus (J.L.D.-R.); jesus.gardeazabalgarcia@osakidetza.eus (J.G.); veronica.velascobenito@osakidetza.eus (V.V.)
2 Biocruces Bizkaia Health Research Institute, 48903 Barakaldo, Spain; rosamaria.izubelloso@osakidetza.eus (R.M.I.); aintzane.apraiz@ehu.eus (A.A.); cristina.penas@ehu.eus (C.P.); ana.sanchezdiez@osakidetza.eus (A.S.-D.); goikoane.canchogalan@osakidetza.eus (G.C.-G.); arrate.sevilla@ehu.eus (A.S.); jesus.cortesdiaz@osakidetza.eus (J.M.C.)
3 Dermatology Service, Basurto University Hospital, 48013 Bilbao, Spain
4 TECNALIA, Basque Research and Technology Alliance (BRTA), 20850 Gipuzkoa, Spain; estibaliz.garrote@tecnalia.com (E.G.); cristina.lopez@tecnalia.com (C.L.-S.)
5 Department of Cell Biology and Histology, University of the Basque Country/EHU, 48940 Leioa, Spain
6 Department of Psychology, Carnegie Mellon University, Pittsburgh, PA 15213, USA; jraserod@andrew.cmu.ed
7 Ibermática Innovation Institute, 48170 Zamudio, Spain; sseijo@ibermatica.com (S.S.); pm.delapena@ibermatica.com (P.M.D.l.P.); ia.cuenca@ibermatica.com (I.C.)
8 Pathology Service, Basurto University Hospital, 48013 Bilbao, Spain
9 Pathology Service, Cruces University Hospital, 48903 Barakaldo, Spain
10 Department of Genetics, Physical Anthropology and Animal Physiology, University of the Basque Country/EHU, 48940 Leioa, Spain; santos.alonso@ehu.eus
11 NorayBio, 48160 Zamudio, Spain; david.fernendez@ekasa.net
12 IKERBASQUE, The Basque Foundation for Science, 48009 Bilbao, Spain
* Correspondence: aintzane.asumendi@ehu.eus (A.A.); lola.boyano@ehu.eus (M.D.B.); Tel.: +34-46-013-034 (A.A.); Tel.: +34-46-015-689 (M.D.B.)
† These authors contributed equally to this work.

Table S1. Summary of the statistical and machine learning studies of risk factors included in the Melanoma Clinical Decision System.

Publications	Methods	Findings	Performance	Data
Mancuso et al. 2020 (9)	Logistic regression, SVM, Decision Tree, Gaussian naïve Bayes classifier (outcome variable: Disease-free vs Melanoma)	Machine learning algorithms classified early-stage melanoma patients with a high or low risk of metastasis. Selected serum cytokines (IL-4 and GM-CSF and DCD) and Breslow thickness were variables that best predicted metastasis.	Accuracy: 80% (Breslow thickness and serum markers model)	Of 448 melanoma patients, 323 were stage I and II patients: 249 disease-free; and 74 developed metastasis.
Penas et al. 2021 (10)	1. Logistic regression (outcome variable: Nevi/Melanoma Diagnosis)<br>2. The Cochran-Armitage Test (RKIP level expression and Breslow thickness)	Higher RKIP protein expression in nevi other than melanomas. Linear increments in RKIP levels correlate with a high probability of the biopsies of being identified as nevus. Higher RKIP protein levels tended to display significantly lower values of Breslow thickness across all AJCC stages.	1. Logistic Regression ($\beta = -2.288$, $q < 0.00$)<br><br>2.Cochran-Armitage test ($q=0.014$)	Of 314 biopsies (239 melanomas and 75 nevi), 92 developed metastasis. Of 171 Stage I and II melanoma biopsies, 33% developed metastasis
Penas et al. 2022 (11) (under revision)	1. Logistic Regression (outcome variable: Disease-free vs Melanoma),<br>2. Cox Regression (outcome variable: onset of melanoma metastasis in months)	Higher levels of Pirin expression are associated with metastatic progression of melanoma, reflected in both a statistically significant increase in the odds and hazard ratios.	1. Logistic regression, ($p=0.007$, OR= 3.851 95%CI [1.453, 10.213], BF10= 11.097).<br>2.Cox analysis: ($p=0.012$, HR=2.305 95% CI [1.203, 4.417], BF10=5.28).	Of 314 biopsies (239 melanomas and 75 nevi): 92 developed metastasis. Of 171 Stage I and II melanoma biopsies, 33% developed metastasis.

Table S2. Summary of the algorithms used in the prognosis module of the Melanoma Clinical Decision System.

Algorithm name	Description
ID3 decision Tree	ID3 (Iterative Dichotomiser 3) is an algorithm used to generate a decision tree invented by Ross Quinlan. ID3 is the precursor of the C4.5 algorithm [42].
CHAID decision tree	The CHAID decision tree algorithm works exactly like the Rapidminer Decision Tree operator with one exception: it uses a chi-squared based criterion instead of the information gain or gain ratio criteria.
Rapidminer Decision Tree	A decision tree is a tree like collection of nodes intended to create a decision on value affiliation to a class using information gain or gain ratio criteria. Decision Trees are generated by recursive partitioning. Recursive partitioning means repeatedly splitting on the values of attributes.
Decision Stump decision tree	The Decision Stump algorithm is used to generate a decision tree with only a single split.
Random Forest decision tree	This algorithm generates a set of a specified number of random trees i.e. it generates a random forest. The resulting model is a voting model of all the trees.
J48 decision tree	Decision tree J48 is the implementation of algorithm ID3 (Iterative Dichotomiser 3) developed by the WEKA [43] project team.
SVM	This operator is an SVM (Support Vector Machine) Learner and it is based on the internal Java implementation of mySVM by Stefan Rueping [44].<br><br>An SVM model is a representation of the examples as points in space, mapped so that the examples of the separate categories are divided by a clear gap that is as wide as possible. New examples are then mapped onto that same space and predicted to belong to a category based on which side of the gap they fall on.<br><br>To keep the computational load reasonable, the mapping used by the SVM schemes are designed to ensure that dot products may be computed easily in terms of the variables in the original space, defining them in terms of a kernel function K(x,y) selected to suit the problem.<br><br>The dot kernel is defined by k(x,y)=x*y i.e. it is the inner product of x and y.<br><br>The radial kernel is defined by exp(-g ||x-y||^2), where g is gamma and it is specified by the kernel gamma parameter. The adjustable parameter gamma plays a major role in the performance of the kernel and should be carefully tuned to the problem at hand.

Table S3. Parameters selected in each algorithm used in the prognosis module of the Melanoma Clinical Decision System.

Decision Trees	Criterion	Gain Ratio
	Minimal size for split	8
	Minimal leaf size	3
	Minimal gain	0.1
	Confidence	0.25
	Maximal depth	5
SVM	C	0
	Convergence epsilon	0.001
	Max iterations	100000

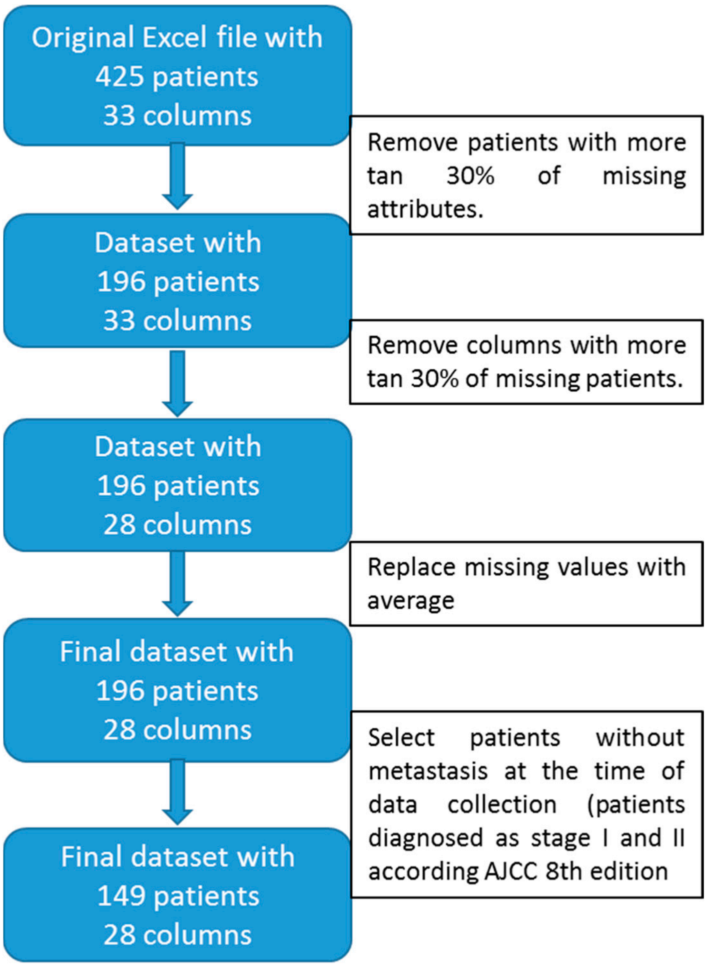

Figure S1. Flow chart of prognosis module.

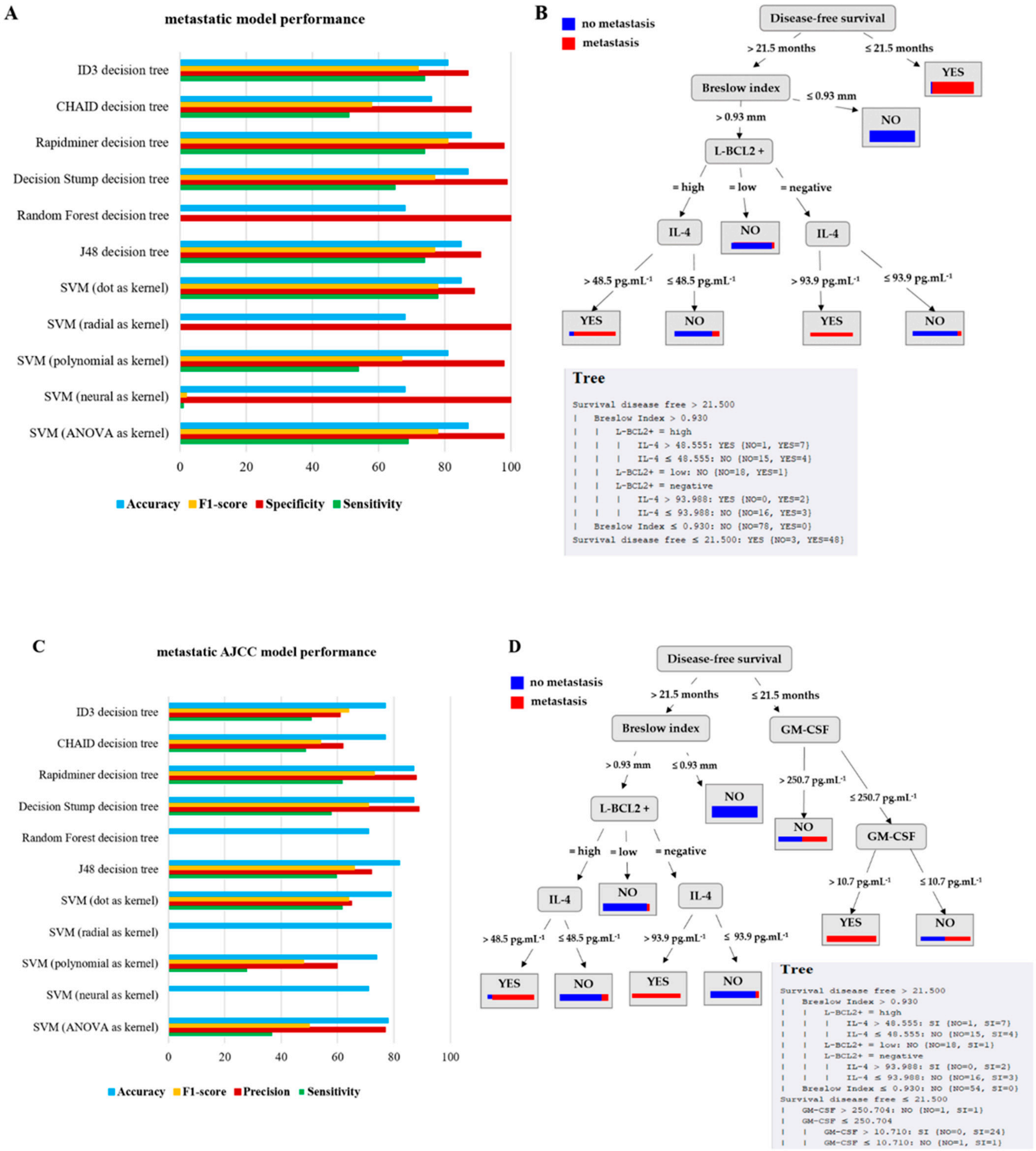

Figure S2: Performance of the models obtained to predict metastasis. A. Performance of the metastasis models obtained from the complete dataset of melanoma patients. B. RapidMiner decision tree model and set of rules applied to the same group of patients (Figures 5A). C. Performance of the metastasis models obtained with the early-stage melanoma patients (stages I and II, according AJCC). D. Rapid/Miner decision tree model and set of rules with the same group of patients as in C (Figure 6A).

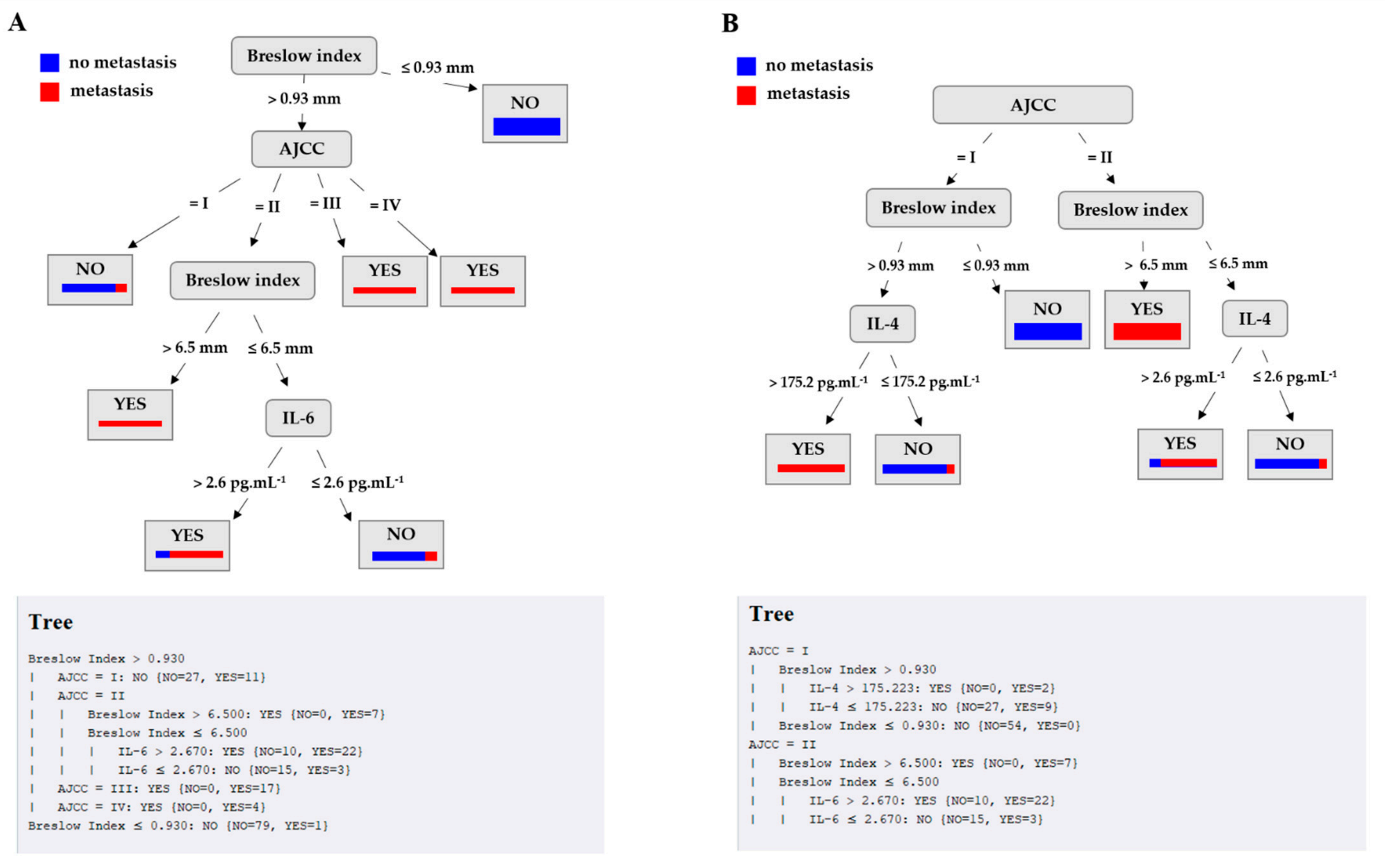

Figure S3. Rapid Miner DT models obtained to predict metastasis in the same group of patients. A. RapidMiner DT model and set of rules applied to the group of patients without DFS attribute (N=196) (Figure 5B). B. RapidMiner DT model and set of rules applied with the early-stage melanoma patients (stages I and II, according AJCC) without DFS attribute (N=149) (Figures 6B).

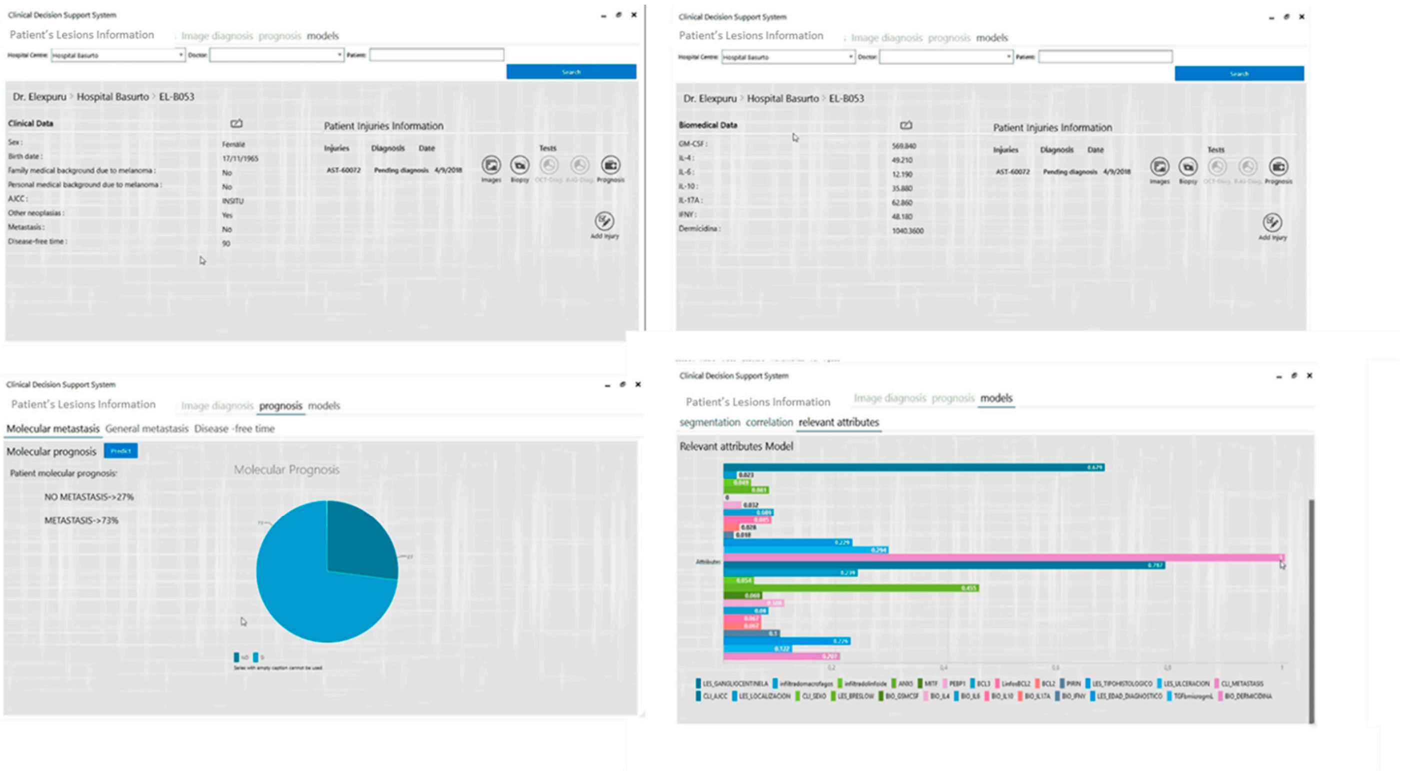

Figure S4: Screenshots from the Melanoma CDSS application.
